# *Primula auriculata *Extracts Exert Cytotoxic and Apoptotic Effects against HT-29 Human Colon Adenocarcinoma Cells

**Published:** 2016

**Authors:** Sahar Behzad, Karim Ebrahim, Mahmoud Mosaddegh, Ali Haeri

**Affiliations:** a *Department of Pharmacognosy, School of Pharmacy, Shahid Beheshti University of Medical Sciences, Tehran, Iran. *; b *Department of Toxicology, School of Pharmacy, Shahid Beheshti University of Medical Sciences, Tehran, Iran. *; c *Traditional Medicine and Materia Medica Research Center, Shahid Beheshti University of Medical Sciences, Tehran, Iran. *; d *Department of Pharmacology, School of Medicine, Shahid Beheshti University of Medical sciences, Tehran, Iran.*

**Keywords:** *Primula auriculata*, cytotoxicity, apoptosis, colorectal cancer

## Abstract

*Primula auriculata* (Tootia) is one of the most important local medicinal plants in Hamedan district, Iran. To investigate cytotoxicity and apoptosis induction of crude methanolic extract and different fraction of it, we compared several methods on HT-29 human colon Adenocarcinoma cells. Cancer cell proliferation was measured by 3-(4, 5‑dimethylthiazolyl)2, 5‑diphenyl‑tetrazolium bromide (MTT) assay and apoptosis induction was analyzed by fluorescence microscopy (acridin orange/ethidium bromide, annexin V/propidium iodide staining, TUNEL assay and Caspase-3 activity assay). Crude methanolic extract (CM) inhibited the growth of malignant cells in a dose-dependent manner. Among solvent fractions, the dichloromethane fraction (CF) was found to be the most toxic compared to other fractions. With double staining methods, high percentage of 40 µg/mL of (CM) and (CF) treated cells exhibited typical characteristics of apoptotic cells. Apoptosis induction was also revealed by apoptotic fragmentation of nuclear DNA and activation of caspas-3 in treated cells. These findings indicate that crude methanolic extract and dichloromethan fraction of *P.auriculata *induced apoptosis and inhibited proliferation in colon cancer cells and could be used as a source for new lead structures in drug design to combat colon cancer.

## Introduction

Colorectal carcinoma, the second most prevalent malignancy and the third leading cause of cancer-related mortalities worldwide (1), is the third in men and forth in women highest cancer mortality in Iran (2, 3). Colon cancer is highly lethal and aggressively malignant due to its dormant course, difficult early diagnosis, metastasis, strong invasion and poor prognosis. Surgical resection remains the only curative treatment for colorectal cancer; however, the outcome is not always satisfactory. Although 70-80% of patients are eligible for curative surgical resection at the time of diagnosis, 50% of all newly-diagnosed patients ultimately develop metastatic disease. Numerous patients should be considered for palliative treatment, including chemotherapy and radiotherapy. However, the toxicity of these chemotherapy medicines to normal tissues and cells has been a major obstacle in successful cancer treatment (4). Over the past decades, the treatment options for colorectal cancer have undergone tremendous changes; however, the clinical results are far from satisfaction (5). Recently, more attention has been paid to naturally occurring chemo preventive compounds that can inhibit, retard, or reverse the process of multistage carcinogenesis with minimal toxicity. Over 60% of the current anticancer drugs have their origin in one way or another from natural sources (6). As a programmed death for cells, apoptosis does not cause lysis in lysosomes and cell membranes, in which there is no efflux of internal contents. This will not trigger inflammation or secondary injuries, which is the ideal result for anti-tumor drugs. Therefore, the search for agents that can trigger apoptosis in tumor cells has become a major goal in anti-cancer drug discovery (7). Apoptosis is characterized by morphological alterations such as cell shrinkage, membrane blebbing, chromatin condensation and DNA fragmentation (8). Morphological hallmarks of apoptosis in the nucleus are chromatin condensation and nuclear fragmentation (9). Recent scientific studies have been focused on herbal medicine as potent apoptotic induction drug candidates (10).

The aim of drug discovery is the ethno medical data approach, in which the selection of a plant is based on information related to its use as a folk medicine. To this end, as parts of our ongoing program of research on the anti-proliferative effects and apoptosis ability of Iranian medicinal plants, the methanolic extract of *Primula auriculata *was tested to suppress colon cancer growth *In-vitro* (11). High cytotoxicity effect of it drew our attention to investigation of the anticancer mechanism of crude methanolic extract and the most effective fraction of *P.auriculata, *using human colon cancer cell lines, HT-29.

The genus *Primula *(more than 400 species) is the most important one in the Primulaceae family, mainly located in the cold regions (12). Some species used traditionally against cough, epilepsy and convulsions (13, 14). Antibacterial, antiviral, antimitotic and anti-inflammatory effects have been also reported for several *Primula* species (14-16). *Primula auriculata *is a valuable local medicinal plant in Hamedan district (locally named Tootia). White powder that was produced by plant inflorescences called Tootia have been used traditionally for eye infectious diseases (17). In Turkey dried herb was sniffed into nose for sneezing to ease respiration in flu (18). Strong antimicrobial and antioxidant activity have been reported from the aerial parts and leaves extract of this plant (19). The present study tries to evaluate *P.auriculata* extracts for investigation of mechanism of cytotoxicity effects on HT-29, a human colon adenocacinoma cell line, extensively used in the study of colon cancer.

## Experimental

*Chemicals*

Dulbecco’s modified eagle medium (DMEM), Fetal bovine serum (FBS), Trypsin (Gibco, USA), (3-[4, 5-dimethylthiazol-2-yl]-2,5 diphenyltetrazolium bromide or MTT, Phosphate Buffered Saline (PBS) salts, Penicillin-Streptomycin, Acridin orange, Ethidium bromide (Sigma-Aldrich, USA), AnnexinV-FITC apoptosis detection kit (Biovision, USA), In Situ Cell Death Detection Kit (Roche, Germany), NucView 488 Caspase-3 assay kit (Biotium Inc., Hayward, USA), Solvents for extraction and fractionation were purchased from Kian Kaveh Azmachem Ltd (Tehran, Iran).

*Plant material*

*Primula auriculata *were collected from Hamedan province of Iran and were identified by botanists at Traditional Medicine and Materia Medica Research Center (TMRC), Shahid Beheshti University of Medical Sciences, Tehran, Iran.

*Plant extraction and fractionation*

The aerial part of plant (100 g) was separated, shade dried and grinded into powder using mortar and pestle at room temperature, then extracted by maceration with methanol for 72 h. The supernatants were filtered using whatman filter paper (No.1), and evaporated under vacuum at temperature below 40°C by means of a rotary evaporator to obtain crude methanolic extract (CM).

**Figure 1:**
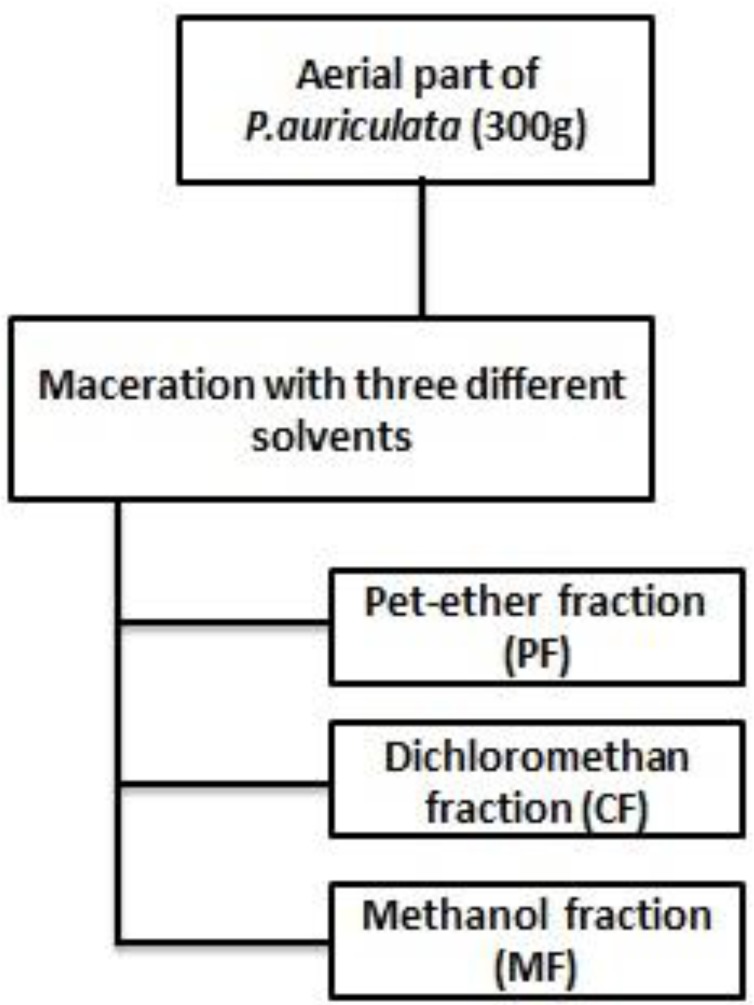
Fractionation scheme of *P.auriculata*

For plant extract fractionation, 300 g of dried powdered plant was macerated with petroleum ether at room temperature; after 72 h, it was filtered, and the filtrate was concentrated as petroleum ether fraction (PF), the residue of the plant was treated with dichloromethan for another 72 h for the dichloromethan fraction (CF) to be prepared, and the steps were repeated for methanol fraction (MF) (Figure 1) (20). The concentrated crude methanolic extract and fractions were then subjected to the cytotoxicity assay.

*Cell line and culture conditions*

HT-29 human colon cancer cells obtained from National Cell Bank of Iran (Pasteur Institute, Tehran, Iran). They were cultured in Dulbecco’s modified eagle medium (DMEM) with 10% Fetal bovine serum (FBS) and were treated with 1% penicillin-streptomycin in a humidified atmosphere with 5% Co_2 _at 37°C throughout the assay.

*MTT assay for assessment of cytotoxicity*

Cell viability was quantified by an MTT colorimetric assay (3-[4, 5-dimethylthiazol-2-yl]-2,5 diphenyltetrazolium bromide assay) (21). HT-29 cells were seeded in 96-well plates at density of 5 × 10^3 ^cells/well and incubated at 37°C. After 24 h of incubation, when cells reached more than 80% confluence, the medium was removed and the cells were treated with fresh medium containing various concentrations of plant extracts to be tested. Untreated cells with 0.05% DMSO (v/v) vehicle served as negative control. After 72 h, the supernatants were removed and a fresh medium containing MTT in PBS (0.5 mg mL^-1^) was added to each well at the time of incubation. After 4 h incubation, the medium containing MTT was carefully removed, and the remaining formazan crystals were dissolved in DMSO. The plates were shaken for 20 min. The absorbance of each well was measured on an enzyme-linked immunosorbent assay reader (TECAN) at the wavelength of 570 nm (22).

*Apoptosis assessment*

To detect the apoptosis induction in HT-29 cells, for each assay, the cells were treated with CM and CF at concentrations required for 50% inhibition of growth of HT-29 cells (IC_50_) for 24 h (40 µg/mL). Untreated cells with 0.05% DMSO (v/v) vehicle served as negative control.

*Acridine orange (AO)-ethidium bromide (EB) double staining cell morphological analysis*

Briefly, at the end of the treatment times, cells were washed with cold PBS and stained with 20 µg/mL of acridine orange (20 µg/mL in PBS) and 20 µg/mL ethidium bromide (20 µg/mL in PBS) just prior to microscopy. A 10 µl aliquot of the gently mixed suspension was placed on microscope slides, covered with glass slips, and examined under an inverted fluorescent microscope (HUND) using a blue filter and photographed with a digital camera (Canon 600D). 300 cells from randomly selected fields were counted and quantified for each data point, in duplicate, for each extract. The cells were scored as viable or dead, and if dead, whether by apoptosis or necrosis as judged from nuclear morphology and cytoplasmic organization. Acridine orange is a vital dye that will stain both live and dead cells, whereas ethidium bromide will stain only those cells that have lost their membrane integrity. Live cells stain uniformly green and can be distinguished from apoptotic cells. Early apoptotic cells will have bright green nuclei with condensed or fragmented chromatin, late apoptotic cells display condensed and fragmented orange chromatin, and cells that have died from direct necrosis have structurally normal orange/red nuclei due to co staining with AO/EB (23). The apoptotic index (percentages of apoptotic cells) was then calculated.

*Annexin V-propidium iodide staining apoptosis test*

The extent of apoptosis and/or necrosis was measured using an annexinV-FITC apoptosis detection kit, by the manufacturers’ recommended protocol. At the end of treatment, cells washed with PBS twice and 100 µl of binding buffer added. The cells were double stained in the dark for 10 min with the fluorescein isothiocyanate (FITC)-labeled annexin V (5 µl) and PI (5 µl) before being analyzed under a fluorescence microscope (24).

*TUNEL assay*

The assay was conducted according to the manufacturer's instructions. Briefly, treated cells were fixed using 4% paraformaldehyde/PBS (pH = 7.4) followed by washing with phosphate buffer saline (PBS) twice. Then the fixed cells were incubated with blocking solution (3% H_2_O_2_ in methanol) for 10 min and rinsed with PBS. Cells were then permeabilized using 0.1% triton X-100in 0.1% sodium citrate. Enzyme solution and label solution reaction mixture were added to label the fragmented DNA at 37°C for 1h using In Situ Cell Death Detection Kit. The FITC-labeled TUNEL-positive cells were imaged by fluorescent microscopy. Changes in the chromatin are accompanied by the introduction of DNA strand breaks into fragments. On TUNEL assay, terminal deoxynucleotidyl transferase labels DNA strand breaks, which catalysis polymerization of labeled nucleotides to free 3´-OH DNA ends in a template-independent manner (25).

*Caspase-3 activity assay*

Apoptosis is mediated by the sequential activation of caspases, which are constitutively present in most cells are inactive pro enzymes. Special roles in this process seem to be played by caspase-3 (26). Caspase-3 (CPP32) is a cytosolic protein that normally exists as a 32 kDa inactive precursor, and is cleaved proteolytically into a heterodimer when the cell undergoes apoptosis (27). Caspase-3 activity was evaluated according to the manufacturer’s instruction of the relevant kit. After treatment of HT-29 cells with 40 μg/mL of the extracts for 24 h, fresh medium or PBS was replaced with ex-medium. The cells were incubated at room temperature for 15-30 min and light protected condition with 5 μl Nuc View 488 substrate solution. For endpoint analysis, cells were washed with PBS and Fluorescence was determined via a florescent microscope using FITC filters, and images were recorded (28).

*Statistical analysis*

The dose–response curves of the compounds were fitted by means of the computer program GraphPad Prism 6.0 (GraphPad Software, USA), and IC^50^ values (the concentration that inhibited 50% of cell growth) were calculated. All *In-vitro* experiments were carried out on three microplates with at least three parallel wells. The analysis of variance (ANOVA) and Dunnett’s post hoc test were used for data analysis. p-values less than 0.05 (p < 0.05) were considered statistically significant.

## Results

*Inhibition of cell viability*

**Figure 2:**
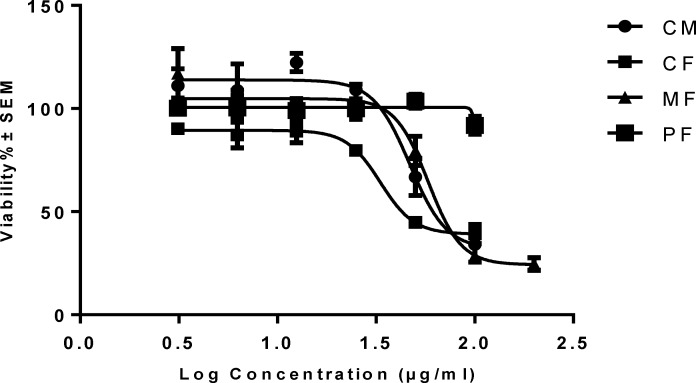
Concentration-viability response curve of crude methanolic extracts (CM) and different fractionsof *P. auriculata* on HT-29 cell lines. Each data is expressed as the mean ± SEM (n = 3).

Inhibition of cell viability caused by crude methanolic extract (CM) of *P.auriculata* and its fractions was examined using MTT assay. In order to compare the cytotoxicity of (CM) of *P. auriculata *and its fractions, the HT-29 cells were incubated with different concentrations for 72 h. The results showed (CM), (CF) and (MF) decreased HT-29 cell viability in a concentration-dependent manner (Table 1and Figure 2). Microscopically, the normal HT-29 cells appeared healthy, polygonal in shape and attached to the well plate. 24 h after addition of (CM), (CF) and (MF) noticeable changes in morphology and density of HT-29 cells were observed.

**Table 1:** IC_50_ values (μg/mL) for crude methanolic extracts (CM) and different fraction of *P. auriculata* on HT-29 cell lines

	**IC**_50_	**IC**_50_ ** range**
Crude methanolic(CM)	43.34	40.36 to 51.56
Petroleum ether fraction (PF)	>100	-
Dichloromethan fraction (CF)	33.20	26.49 to 41.61
Methanol fraction (MF)	58.05	46.71 to 72.14

**Figure 3:**
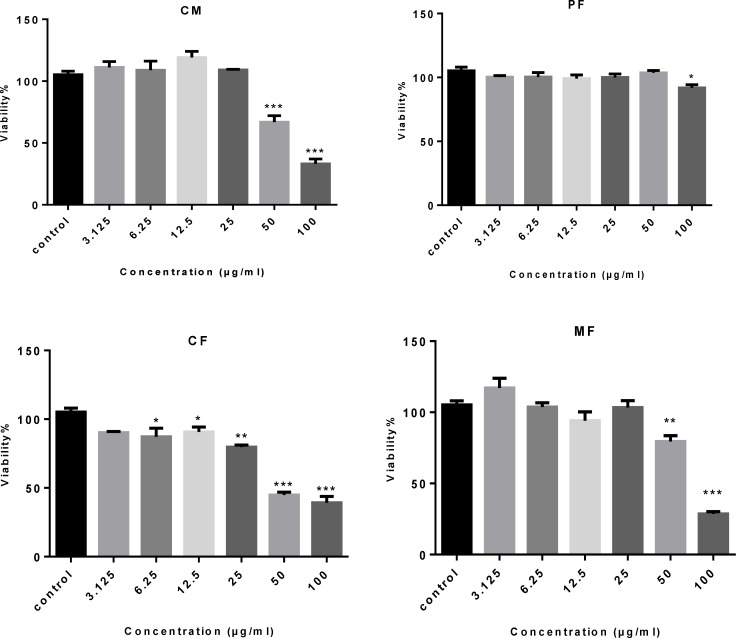
The cytotoxic effect of crude methanolic extract (MC) and different fractions of *P. auriculata* on HT-29 cell lines. Results are mean ± SEM (n = 3). *p < 0.05, **p < 0.01 and ***p < 0.001 compared to control

*Apoptosis assessment*

*In-vitro* cytotoxic evaluation of *P.auriculata *fractions demonstrated that dichloromethan fraction (CF) exhibited the most cytotoxic effects. Then the crude methanolic extract (CM) and dichloromethan fraction (CF) were selected to investigate apoptosis induction ability. Apoptosis was induced by treating HT-29 cell with (CM) and (CF) at their IC_50 _values approximately (29, 30). 

*Acridine orange (AO)-ethidium bromide (EB) double staining cell morphological analysis*

**Figure 5:**
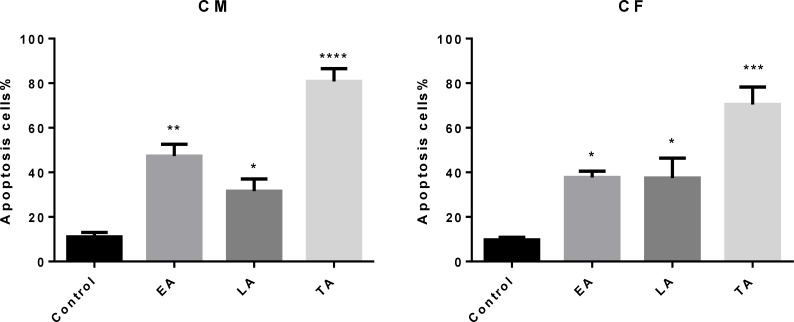
Comparison of the number of apoptotic cells (Apoptotic Index) scored by fluorescence microscopy using acridine orange/ethidium bromide (AO/EB). (EA) early apoptosis, (LA) late apoptosis and (TA) total apoptosis. An increase in the number of apoptotic cells was observed in (CM) and (CF)-treated HT-29.Results are mean ± SEM (n = 3). *p < 0.05, **p < 0.01, ***p < 0.001 and ****p < 0.0001 compared to control. A minimum of 300 cells was counted in every sample

The results from AO/EB double staining are shown in Figure 4. From the data it was clear that in 40 µg/mL of (CM) and (CF), the number of viable cells decreased tremendously. Besides some cells exhibited typical characteristics of apoptotic cells like plasma membrane blebbing. However the number of cells stained red did not increase. This indicates the most of the cells were not undergoing necrosis and cell death occurred primarily through apoptosis. In Figure 5, results of early apoptotic (EA), late apoptotic (LA) total apoptotic (TA) and negative control cell population were expressed as percentage of apoptosis.

**CBFigure 4:**
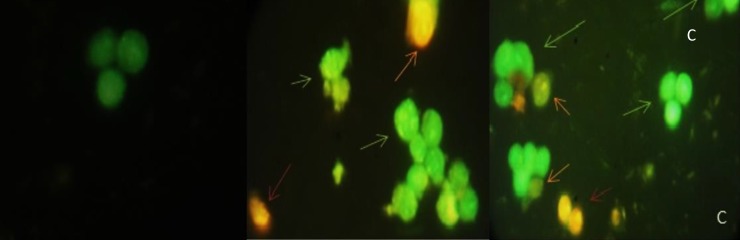
Acridine orange/ethidium bromide staining of HT-29 cells to detect apoptosis induced by different extract of 40 µg/mL of (A) negative control (B) CM(C) CF. Live cells are uniformly green, whereas apoptotic cells (green arrows for early apoptosis (EA), orange arrow for late apoptosis (LA) and apoptotic bodies) characterized by shiny green (EA) and yellow-orange staining (LA) due to chromatin condensation and loss of membrane integrity. Red arrow for necrosis. Magnification400

*Annexin V-propidium iodide staining apoptosis testing*

**Figure 6:**
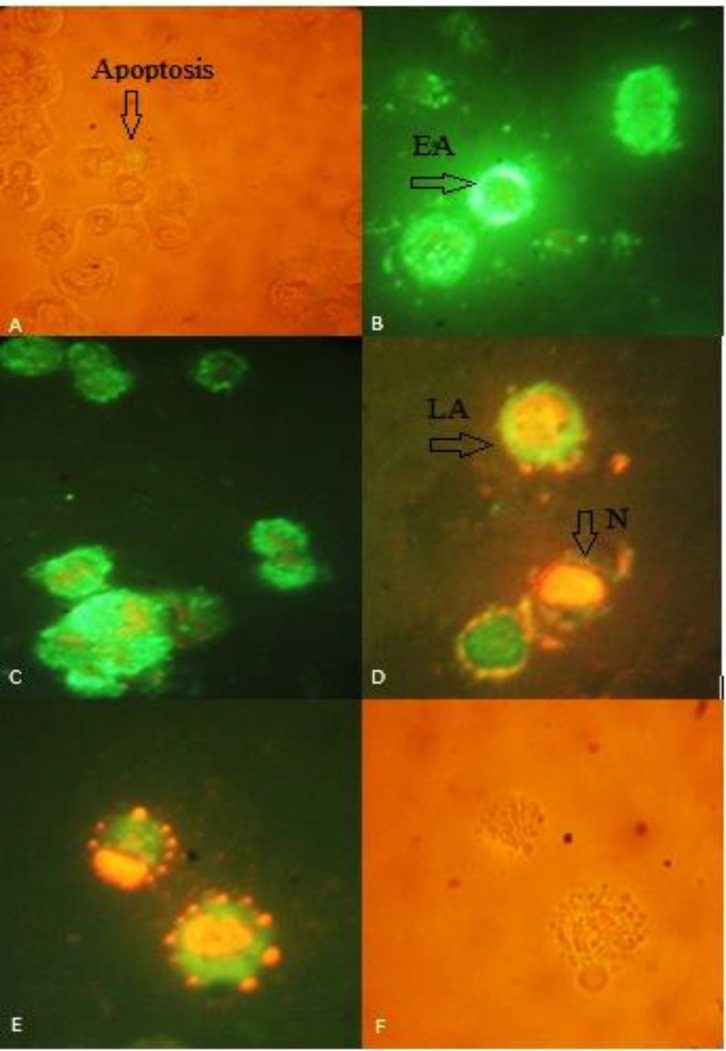
Annexin V-propidium iodide staining apoptosis test of HT-29 cells to detect apoptosis induced by different extract of 40 µg/mL of (A) negative control in light microscopic view (B) CM (C) CF (D) early apoptotic(EA), late apoptotic (LA) and necrosis (N) in CF (E,F) fluorescent view and light microscopic view of apoptotic bodies, respectively. Green annexin fluorescence marks cells with loss of membrane asymmetry as indicator of membrane damage. Necrotic cells show red PI staining of nuclei. Loss of integrity of nuclear envelope and formation of peripheral, sharply delineated masses of condensed chromatin or apoptotic bodies are visualized. Images were taken 400

Phosphatidylserine (PS) translocation from inner part of plasmamembrane to outer part is believed to be an early event in apoptosis. Binding of Annexin V to phosphatidylserine in presence of calcium ions results in green fluorescence. During late apoptosis or necrosis, owing to increased membrane permeability, PI also enters the cell and binds to cellular DNA, staining the nucleus red. different labeling patterns in this assay enabled us to identify different cell populations: live cells (Annexin V-FITC negative/PI negative), early apoptotic cells (the intactness of the cell membrane, affinity for annexin V-FITC and devoid of PI staining), late apoptotic/necrotic cells (the cell membrane loses its integrity, the cell becomes both annexin V-FITC and PI staining) and dead cells (Annexin V-FITC negative/PI positive). Annexin V/propidium iodine staining test revealed typical apoptotic phenotype in cells treated with 40 µg/mL of (CM) and (CF) for 24 h (Figure 6), in contrast, control cells without treatment displayed a small quantity of apoptosis.

The number of early apoptotic cells decreased in 40 µg/mL of (CF). However the number of necrotic cell did not increase, Induction of apoptosis is dominant. 

*TUNEL assay*

**Figure 7:**
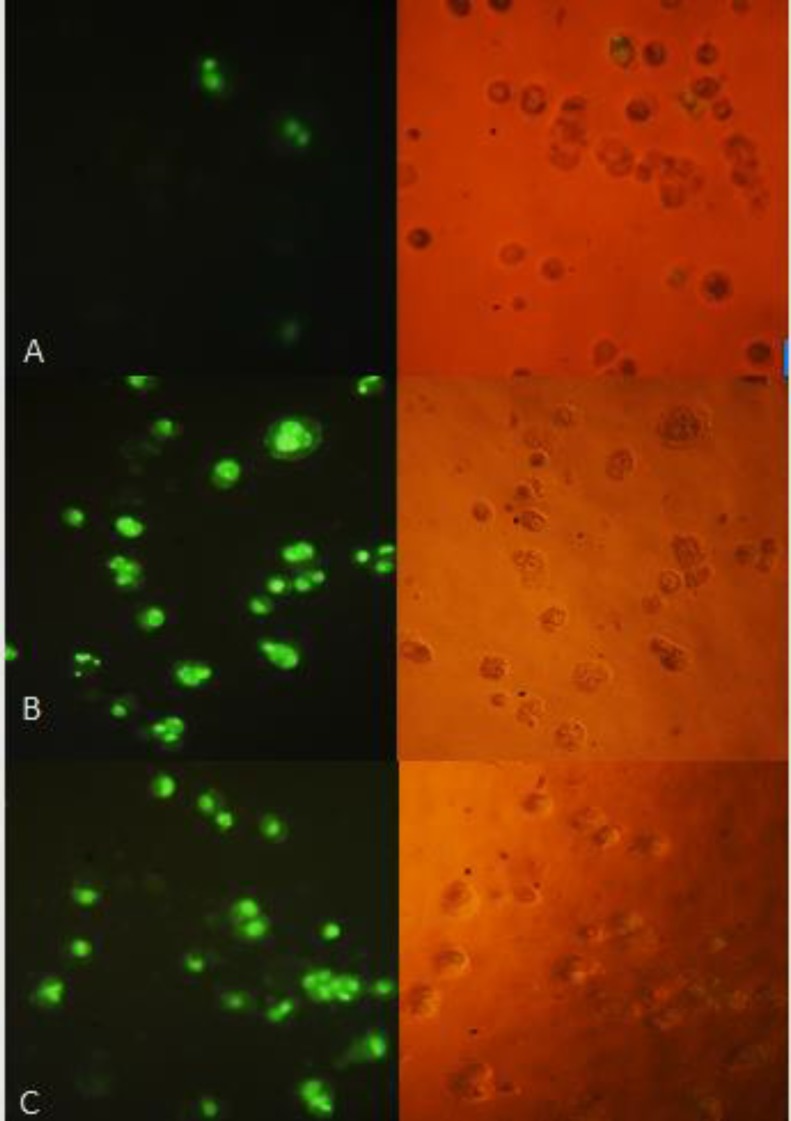
TUNEL assay: HT-29 cells were treated with 40 µg/mL of (A) control (B) CM (C) CF. The right side figure remarks the light microscope view and the left side remarks fluorescent view of the same field. The level of staining indicates the degree of DNA damage induced by treatment, where more positively stained cells are in the final stages of apoptosis. Magnification: 200 ×.

The cell apoptosis was observed after TUNEL staining in Figure 7, which reveals the apoptotic fragmentation of nuclear DNA. The cells of the negative control group appeared normal, whereas treated cells with 40 µg/mL of (CM) and (CF) in bright green exhibited significant apoptosis.

*Caspase-3 activity assay*

**Figure 8:**
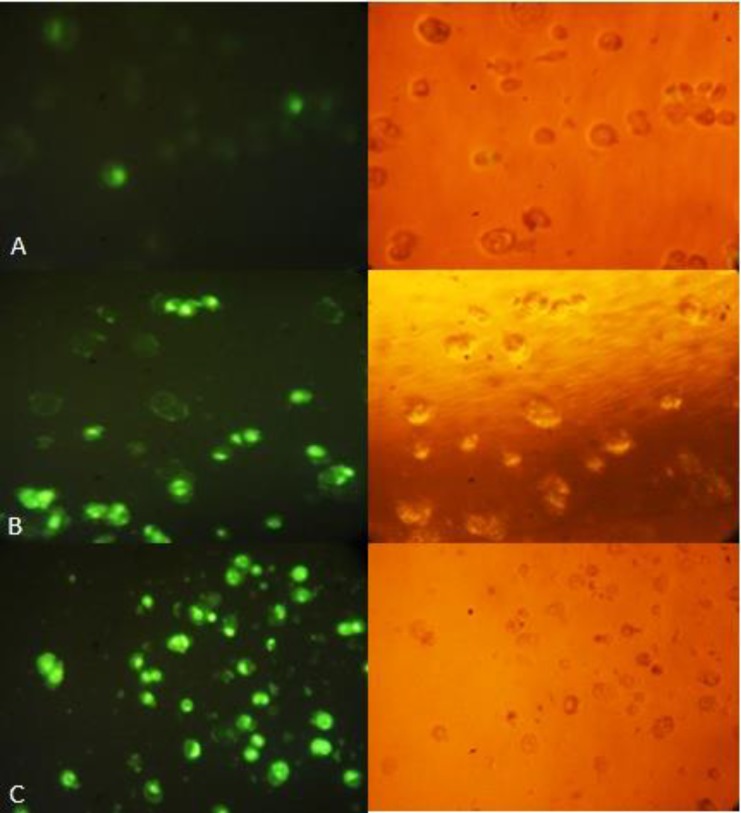
Caspase-3 apoptosis assay shows increased apoptosis in HT-29 cells treated with CF (40μg/mL/24 h). Nuclear DNA of apoptotic cells were stained green by the enzymatically released DNA dye. (A) Control, (B) CM, (C) CF. The right side figure remarks the light microscope view and the left side remarks fluorescent view of the same field. Magnification: 200×.

In apoptotic cells, caspase-3/7 activity cleaves the substrate, releasing the high affinity DNA dye; which immigrates to the cell nucleus and stains DNA with bright green fluorescence. In this study, the treated apoptotic cells showed, as indicated in Figure 8, a significant increase in the activity of caspase-3 after 24 h of exposure to (CF) than (CM), indicating that extracts-induced apoptosis is caspase-3 dependent.

## Discussion

Apoptosis is a programmed cell death and a highly organized physiological mechanism to destroy injured or abnormal cells (31) and it is an attractive screening endpoint in anticancer drug discovery projects. It is induced by many clinically used and effective anticancer agents. In addition, it seems logical that by screening for apoptosis, agents that are cytotoxic by unspecific mechanisms will be excluded (32). A wide variety of natural substances have been recognized to have the ability to induce apoptosis in various tumor cells of human origin (33, 34). These substances are compounds with different chemical entities and many of them are present in plants with medicinal value and in various fruits and vegetables, commonly consumed by humans. So it is important to screen apoptotic inducers from plants, either in the form of crude extracts or as components, isolated from (35). *P. auriculata *is rich in phenolic compounds, flavonoids, saponins and glucosinulates in preliminary tests (19). These compounds have many pharmacological and biological activities including antioxidative (36), anti-inflammatory and anti-tumor ones (37, 38). Primin (2-methoxy-6-n-pentyl-1, 4-benzoquinone), from *P. obconica* has shown antimicrobial and antitumor properties (13). Also 5 known flavones from *P.denticulata* induced apoptosis and possessed strong cytostatic properties even at low concentrations on HL-60 cell (39). In this study, the IC_50_ values of the fractions and methanol extract of *P. auriculata *have been obtained from MTT assay were compared in Table 1.An active constituent (s) of intermediate polarity as seen in other species is likely caused by the observed cytotoxicity, so future bioassay guided fractionation is needed for focusing on CF.CF and CM fractions can serve to trace the highly active phytochemicals. Considering the chemo-physical properties of different compound and solvent polarity, it is possible to predict the cytotoxic compounds or the compounds with the maximum effect which might be present in CF and CM*. *The CF fraction as the most cytotoxic fraction and CM extract, were analyzed to establish its apoptotic activity in the HT-29 cell line.

There are many ways of detecting apoptotic cell death *in situ* and *In-vitro*. Utilization of fluorescence microscope in apoptosis detection approaches has a number of significant advantages. First, fluorescence generally ensures a higher signal-to-noise ratio than chromogenic techniques, which improves sensitivity. Second, fluorescent dyes and fluorescent fusion proteins can be used. Finally, the detection method does not involve an enzymatic reaction, whose efficacy may be affected by several variables including buffer composition, pH and temperature (40). Double staining methods for detecting apoptosis (AO/EB and Ann V/PI), provide reliable and reproducible results, so, distinguish clearly subpopulations of apoptotic cells (early or late apoptotic cells). The results of apoptotic cell subpopulations which identified by the AO/EB and Ann V/PI methods were highly reproducible (41). In this study, as shown, TA and EA cell population in CM treated cells were more than CF but, LA cell population in CM treated cells were less than CF in AO/EB staining assay, means the most frequent number of apoptotic cells observed in 40 µg/mL CM treated cells and early apoptosis stage cells were significantly increased. CM and CF also increased the amount of cells apoptosis (early and late stages) via (PS) translocation, which is an early event in apoptosis. The DNA breaks occur before changes in cell morphology and the TUNEL assay which is specific and apoptosis hallmark, can be applied to study the early events of apoptosis (42, 43).This technique is characterized by higher sensitivity among common cyto (histo) chemical approaches and has long been considered to be the gold standard to detect apoptosis in situ. Both CM and CF showHT-29 cell death via DNA fragmentation, but it could not be possible to differentiate apoptotic cells from necrotic ones. Procaspase-3 is a protease which plays significant role in apoptosis in various different cell types. Procaspase-3 is activated by different stimuli inducing apoptosis called cleaved or activated caspase-3. The detection of the cleaved form of caspase-3 provides a unique, direct and sensitive method which is specialized for determination of apoptosis in cells. As seen, Apoptosis was detected in HT-29 cell when treated with CM and CF and Figure 8 shows the green shiny fluorescence causedbyactivatedcaspase3inthe condensed nuclei which lead to simulate the spread of cell apoptosis signals. The light microscopic view in Figures 6, 7 and 8 certified the apoptosis induction by morphological changes such as pyknotic nuclei, membrane blebbing and swollen cytoplasm. All of these methods have their drawbacks and the best policy for apoptosis verification is to employ a panel of different mechanistic approaches (44). In conclusion, to the authors’ knowledge, for the first time, this study demonstrated that crude methanolic and CH_2_Cl_2_ fraction of *P. auriculata* exhibited a potent inhibitory effect on colon cancer cell growth, which was mediated by its pro-apoptotic and antiproliferative activity. Besides, the ethno botany data of this species, which is used for eye infection, and antimicrobial agent, cytotoxicity of other species of this genus, led us to test this medicinal plant. Further studies are required to assess molecular mechanism and other pathway for apoptosis. These results provide a strong scientific foundation for the development of novel anticancer agents from the bioactive ingredients in the most effective fraction.
